# Supplementary material for: Birth in shelters: Midwives’ lived experiences in providing childbirth care amidst war in Gaza
Source: PLoS One. 2026 May 20;21(5):e0339551. doi: 10.1371/journal.pone.0339551 (PMC13189299; doi:10.1371/journal.pone.0339551)
Supplement: S1 Appendix — (DOCX) [file pone.0339551.s001.docx]

S1 Appendix: Example of data extract containing units of data and line-by-line coding

| **Data extract units of data** | **Early descriptive codes/line-by-line** |
| --- | --- |
| Midwife#4  Of course, transportation was difficult, and mobile communication was difficult (no signal), so it was hard to contact anyone, even the ambulance. At around 1 a.m., a man came to my tent saying: my wife told me that she was in a lot of pain. | No transportation. No signal for mobile communication  No ambulance,  Requesting assistance from the MW. People trust the MW. Woman in labor |
| Midwife#7  She was displaced and staying at her relatives' house when she suddenly went into labor at night. People didn't know that there is a midwife nearby, but our neighbors told them, and they came to me at around 3 a.m. To be honest, I didn't have all the necessary tools to deliver the baby. We tried to deliver the baby with the minimal available resources we had. Thank God, the delivery went smoothly. The baby was a girl. para1 previous 1 The delivery went well, thank God. I followed up with her until she was doing well and breastfeeding. But we were in the worst possible situation. It was a difficult birth... no electricity, no... we used a flashlight. We delivered the baby safely as best we could, meaning we tried to work with the resources we had and the tools available. | Displaced woman.  Neighbors informed about the MW. People trust and request care from midwife.  No delivery kits. Lack of essential tools. Lack of preparedness. Midwife the only available caregiver. Unexpected birth  Successful outcomes  No electricity, used flashlight  Minimal resources  Birth in shelter |
| Midiwfe#9  I wrapped it in a blanket and took the blanket I had taken from the street and my aunt gave me a towel, and I put it underneath because it is clean, then I added the blanket on top of it. There were no clothes. | No blanket for the newborn.  No clothes  Newborn care  Birth in the street |
