# Supplementary material for: Birth in shelters: Midwives’ lived experiences in providing childbirth care amidst war in Gaza
Source: PLoS One. 2026 May 20;21(5):e0339551. doi: 10.1371/journal.pone.0339551 (PMC13189299; doi:10.1371/journal.pone.0339551)
Supplement: S2 Appendix — (DOCX) [file pone.0339551.s002.docx]

S2 Appendix: Example of development from colliding codes to sub-themes and themes

| **collated data** | **initial-themes** | **Theme** |
| --- | --- | --- |
| Lack of equipment  No delivery kits  Lack of preparedness  Improvised use of house/kitchen utensils  No transportation  Lack of sanitation and antiseptic materials  No medications  Dangerous and life-threatening environment  Scared and frightened from bombing but determined to help  Chaotic scenes and sounds  Refugees and shelters attacked, no safe places  Hospitals attacked, no safe places to work | Resource constrains, lack of preparedness, lack of supplies | Unprotected in ruthless warfare |
| Midwife's family responsibility  Displaced multiple times  Family's Health Impact on Work  Partial irregular salary  Constant on duty  Giving the best at work despite hardship  Pouring all energy in work and family to prevent mental trauma | Midwives balancing personal and working life during war | Midwives’ Roles and Emotional Experience |
