# Supplementary material for: Birth in shelters: Midwives’ lived experiences in providing childbirth care amidst war in Gaza
Source: PLoS One. 2026 May 20;21(5):e0339551. doi: 10.1371/journal.pone.0339551 (PMC13189299; doi:10.1371/journal.pone.0339551)
Supplement: S3 Appendix — (DOCX) [file pone.0339551.s003.docx]

**S3 Appendix: Interview guide (in English)**

Interview Guide

During the interview:

Please obtain the following information from each participant

Basic information about each participant: Age, place of residence before the war, current place of residence, work/job.

Professional background: level of education (Bachelor/Master), place of graduation (name of university), year of graduation.

Experience: Years of experience as a midwife?

Questions about the experience of helping pregnant women to give birth in a shelter or at tent during the war.

Can you tell me about your personal experience providing care to women giving birth in a shelter or tent during the war?

(Examples of questions for help: When did it happen? How were you reached for help? Where was it done? What did you do? How did you prepare for the woman before giving birth? How did you manage? How did you assist the newborn? What kind of equipment did you have? Where did you get it? What was it? Did you have short of equipment? What would kind of equipment and support would you recommend to make available for midwives in this situation?

Who helped you during the birth? How do you feel now about this experience? What difficulties did you face? What challenges did you encounter while helping women give birth in shelters during the war? Can you explain more? Can you give me an example?

How has the war affected you as a midwife? Your workplace? Your place of residence? You family? Mobility and transportation? Working hours? Give me an example?

Since the beginning of the war: When was the last time, you received a salary/amount of money to live on? Why do you continue to work without a salary? What motivates you.

Mental and physical health for you and your family? Is anyone in your family affected by the war? Have you lost someone?

Morale? Can you describe your energy, enthusiasm and passion of performing your role? Can you describe the struggle that you pass through the war, how it effects on your physical and psychological status?
