# Supplementary material for: Birth in shelters: Midwives’ lived experiences in providing childbirth care amidst war in Gaza
Source: PLoS One. 2026 May 20;21(5):e0339551. doi: 10.1371/journal.pone.0339551 (PMC13189299; doi:10.1371/journal.pone.0339551)
Supplement: S4 Appendix — (DOCX) [file pone.0339551.s004.docx]

**S4 Appendix:** **Informed Consent Form (in English)**

**Consent Form for Participation in Scientific Research**

**Dear Midwife,**

We are researchers from Gaza and the West Bank exploring the experiences and insights of those who assisted pregnant women during childbirth in shelters or tents for the displaced in Gaza during the war.

We would like to invite you to participate in the study so that we can ask questions and gain a better understanding of this matter.

Your participation is voluntary, and your information will be kept confidential. No names will be used during the analysis or results. You are free to refuse to participate or not to answer a specific question. Refusal or withdrawal will not affect you in any way and will have no consequences.

There are no personal benefits for you in participating, but your contribution is crucial in helping us convey the voice, experiences, and sacrifices of individuals like XXX in Gaza, who assisted pregnant women during childbirth outside hospitals during the war. This information will be valuable in offering guidance on essential skills and competencies needed by individuals working in politically conflicted regions to provide safe reproductive health services to women in peace and wartime.

We ask your permission to record the interview, to ensure that your opinions are accurately captured. The recordings will be transcribed to text without any identifiable information. We assure you that the recordings will be encrypted and stored safely accessible only by the two Arabic speaking researchers. The recordings will be destroyed once this research is completed. If you wish, we can share the results of this research with you and inform you of any dissemination actions taken based on the findings.

The interview will take approximately 45-60 minutes.

Do you have any questions?

If you consent to participate in this study, please give your oral consent in the beginning of the interview by confirming that you have read this information and are aware of your right to withdraw, and that you voluntary want to participate.

Do you want to participate in this study?

If you have other questions or complaints, you are welcome to contact the researcher: XXX
