# Supplementary material for: Birth in shelters: Midwives’ lived experiences in providing childbirth care amidst war in Gaza
Source: PLoS One. 2026 May 20;21(5):e0339551. doi: 10.1371/journal.pone.0339551 (PMC13189299; doi:10.1371/journal.pone.0339551)
Supplement: Attachment — Submitted filename: Response to reviewers.docx [file pone.0339551.s007.docx]

**Point-by-point response to Editor comments**

Journal Requirements:

When submitting your revision, we need you to address these additional requirements.

**1. Please ensure that your manuscript meets PLOS ONE's style requirements, including those for file naming. The PLOS ONE style templates can be found at**

<https://journals.plos.org/plosone/s/file?id=wjVg/PLOSOne_formatting_sample_main_body.pdf> and

[https://journals.plos.org/plosone/s/file?id=ba62/PLOSOne_formatting_sample_title_authors_affiliations.pdf](https://track.editorialmanager.com/CL0/https:%2F%2Fjournals.plos.org%2Fplosone%2Fs%2Ffile%3Fid=ba62%2FPLOSOne_formatting_sample_title_authors_affiliations.pdf/1/010f019d61fd6eed-164b2448-0e93-4173-a257-2105e454ca92-000000/3yESaeUyMG6sX7I8yE5bx9HiJPMeZjiwj6CWGhkhH2w=257)

Response: The title page was revised according to the journal style.

The body of the manuscript was formatted as per the journal guidelines.

**2. In the ethics statement in the Methods, you have specified that verbal consent was obtained. Please provide additional details regarding how this consent was documented and witnessed, and state whether this was approved by the IRB**

Response: Page 8 (lines 252-256): under Ethical consideration, we added the following details regarding how the consent was documented and witnessed:

*“A written consent statement was included on the first page of the interview guide and was read by all participants, with approval provided by each midwife. At the start of recording, the interviewer (second author) read the consent aloud, and each midwife gave recorded oral approval before the interview commenced.”* **Modified.**

Page 8, lines 263-265: there is a statement regarding ethical approval by the IRB and the number of the approval. **No further modification needed.**

**3. Please include a complete copy of PLOS’ questionnaire on inclusivity in global research in your revised manuscript. Our policy for research in this area aims to improve transparency in the reporting of research performed outside of researchers’ own country or community. The policy applies to researchers who have travelled to a different country to conduct research, research with Indigenous populations or their lands, and research on cultural artefacts. The questionnaire can also be requested at the journal’s discretion for any other submissions, even if these conditions are not met.  Please find more information on the policy and a link to download a blank copy of the questionnaire here:** [**https://journals.plos.org/plosone/s/best-practices-in-research-reporting**](https://journals.plos.org/plosone/s/best-practices-in-research-reporting)**. Please upload a completed version of your questionnaire as Supporting Information when you resubmit your manuscript.**

Response: A completed copy of the PLOS questionnaire on inclusivity in global research will be uploaded as supporting information with the revised manuscript.

4. PLOS requires an ORCID iD for the corresponding author in Editorial Manager on papers submitted after December 6th, 2016. Please ensure that you have an ORCID iD and that it is validated in Editorial Manager. To do this, go to ‘Update my Information’ (in the upper left-hand corner of the main menu), and click on the Fetch/Validate link next to the ORCID field. This will take you to the ORCID site and allow you to create a new iD or authenticate a pre-existing iD in Editorial Manager.

Response: I attempted to update my ORCID iD in the system; however, this was not possible. It appears that my ORCID iD is already linked to my account in the PLOS ONE system as a reviewer, and the two accounts are not synchronizing properly. I would appreciate your technical assistance in resolving this issue.

[Sahar Hassan (0000-0002-6887-9993) - ORCID](https://orcid.org/0000-0002-6887-9993)

**5.  Your ethics statement should only appear in the Methods section of your manuscript. If your ethics statement is written in any section besides the Methods, please delete it from any other section.**

Response: The ethics statement is included only in the Methods section.

**6. If the reviewer comments include a recommendation to cite specific previously published works, please review and evaluate these publications to determine whether they are relevant and should be cited. There is no requirement to cite these works unless the editor has indicated otherwise.** 
Response: Thank you for the note. Well noted.

**----------------------------------------------------------------------------------------------------**

**Point-by-point response to reviewers’ comments**

**Reviewers' comments:**

**Reviewer #1:** The article is well written and methodologically clear.

**I found the themes could be better titled for example Theme 1: Unprotected in ruthless warfare could be Midwifes Safety or something similar.**

Response: Thank you for this valuable suggestion. We appreciate the recommendation to simplify theme titles for clarity. While we agree that titles such as “Midwives’ Safety” are more concise, we intentionally retained “Unprotected in ruthless warfare” to reflect the intensity and lived experiences expressed by midwives.

The analytic process involved extensive and iterative discussions among the three authors, culminating in consensus on theme names that are both conceptually grounded and contextually rich. The authors were deeply immersed in the data over several months, engaging in repeated reading, re-reading, and collective reflection on midwives’ narratives.

We aimed for the theme titles to capture not only issues of safety, but also what midwives experienced and expressed in their own terms. The notion of being “unprotected” while providing care to pregnant women emerged strongly from the data and conveys the reality participants sought to communicate. In this context, midwives described working in an environment where safety could not be assured, even while delivering essential care. For these reasons, we respectfully prefer to retain the original theme titles, as they were derived through a rigorous and collaborative analytic process and are intended to faithfully represent participants’ voices and the depth of the context.

**There is a known war that the authors refer to but there is often a tendency to add adjectives to explain the brutality of the war e.g. ruthless war etc. I would advise the authors to consider reframing the war without adjectives.**

Response: Thank you for this comment. We acknowledge that this war has been experienced by midwives as exceptionally severe and devastating, with widespread impact on civilians, including women and children. One of the authors (the second author) has lived through these conditions daily, which inevitably shaped the research context and the team’s engagement with the data.

We recognize the importance of maintaining academic rigor and minimizing bias. At the same time, our use of descriptive language was grounded in participants’ narratives and intended to faithfully reflect their lived experiences. We have therefore aimed to balance neutrality with an accurate representation of the intensity and context conveyed by these midwives.

However, we have revised the term “war” throughout the manuscript and removed unnecessary adjectives (lines 83, 298, 575, 594, 597, 635, 646, and 674). However, we retained the adjective in the first theme, as we consider it essential to faithfully reflect midwives’ voices and the contextual depth. **We hope this satisfies the reviewer’s inquiry. Modified.**

**Moreover, as qualitative researchers, if there are any prior experiences or biases that may influence the interpretation of the results, that needs to be mentioned. This is often encouraged in any qualitative research.**

**Response:** Thank you for the comment. We have reflected on the author's previous experiences, and elaborated on credibility and dependability (page 6, lines 192-196) and (page 8, lines 234-236). **No modifications were needed.**

**The second theme has a very large results description, which either needs to be shortened or another sub-theme introduced.**

Response: Thank you for the suggestion. The second theme included five subthemes related to the professional role and associated emotional tensions, which were presented sequentially and supported with appropriate quotations. These subthemes were: midwifery practice and confidence, improvised preparedness, accessibility and availability among the displaced, balancing personal and professional life during war, and midwives’ emotional strength and faith.

Our initial analysis generated 6–8 themes. However, through iterative revisions, in-depth discussions, and extensive conceptual work among the three authors, we reached a consensus to consolidate these into five themes and ultimately into three overarching themes. Arriving at these three themes required substantial analytical effort to ensure they were comprehensive while avoiding redundancy.

The presentation of Theme 2 follows these five subthemes, with italics used at the beginning of each paragraph to signal a new subtheme while maintaining a coherent narrative flow. With respect to the reviewer’s suggestion, we prefer to retain the current structure to avoid redundancy and unnecessary repetition across themes. **No modifications were needed.**

**Do the midwives receive any form of prior mental health training to provide support given there is a history of conflict in the area being served?**

Response: Thank you for this question. In general, training for healthcare providers is largely dependent on the availability of external funding, and there is no institutionalized in-service training in either the West Bank or Gaza. In Gaza, midwives working in primary healthcare clinics have received some training in mental health; however, midwives working in hospitals do not receive such training, as available funding is primarily directed toward primary healthcare settings. **No modifications were needed.**

**Lastly, I wondered why the midwives didn't share more insights of birth complications example PPH or eclampsia or obstructed labour as a challenge in the circumstances.**

**Response:**  Thank you for this important note. During the research team discussions, we also reflected on why midwives did not share situations involving complications. We believe that midwives may have been reluctant to disclose such cases, as assisting women to deliver outside the hospital is considered an illegal practice, and midwives are not permitted to provide this type of care even in a crisis situation, as there is no policy in place. Consequently, they may have feared potential repercussions or sanctions from the health system. It is therefore possible that midwives selectively shared narratives that ended without complications.

Notably, two midwives reported assisting a labouring woman with history of infertility and a previous cesarean section, as the war was intense and both cases had no access to an ambulance to be transferred to the hospital. We did include relevant quotations from midwives (MW7 and MW8; page 18, lines 546–551, and page 19, lines 556-558), who reported assisting women with potential risks. Similarly, MW8 reported assisting a woman with a prior history of postpartum hemorrhage at a focal medical point in a school, where she was the most qualified provider and ambulance access was not possible. In this situation, Pitocin was administered intravenously, as it was available at the site. We also acknowledged this in the limitations section (page 21, lines 659-662) and on (page 21, line 662-666), we added the following:

*“Additionally, midwives may have been reluctant to disclose cases with complications, as assisting women to deliver outside the hospital is considered an illegal practice. Consequently, they may have feared potential consequences or sanctions from the health system. It is therefore possible that midwives selectively shared narratives that ended without complications.”*

**We hope this elaboration and modification satisfy the reviewer’s comment. Modified.**

**The results and discussion expand on lack of services which gives the reader a sense of the challenges but I wondered if the authors can comment on this query?**

Response: Thank you for this query. Displaced populations were indeed left without adequate services, as the health system is primarily structured around hospitals and primary healthcare clinics provided by the two main sectors: the Ministry of Health and UNRWA. During periods of intense airstrikes and evacuation orders, UNRWA clinics, despite being widely distributed, often shut down. Access to hospitals was also severely constrained due to a lack of transportation, destruction of roads, and ongoing hostilities.

Within displaced communities, some grassroots groups and local NGOs established focal medical points, typically run by members of the affected communities. Many of these points operated with few or no physicians, nurses, or midwives, and were equipped only with basic supplies such as cotton, gauze, antiseptics, common analgesics, and occasionally antibiotics.

Midwives were still required to attend their scheduled hospital shifts despite the risks. Some reported walking for hours under active airstrikes to reach their workplaces, as they had no alternative if they wished to retain their positions and secure a portion of their salary to support their families. Outside their official working hours, particularly in the evenings and at night, midwives were often approached by pregnant women within their communities for assistance.

A small number of midwives reported receiving one or two disposable delivery kits, assembled and distributed by local NGOs; however, this was insufficient, and many midwives did not receive any kits at all. **We hope this elaboration clarifies the reviewer’s query. No modifications were needed.**

---------------------------------------------------------------------------------------------------------

**Reviewer #2:** Thank you, editorial team, inviting me to review this manuscript. This is a methodologically sound and ethically important qualitative study. The authors present a compelling narrative that is both scientifically rigorous and deeply human. The findings have critical implications for humanitarian policy, midwifery education, and the protection of healthcare workers in conflict zones. I have several comments and questions aimed at strengthening the manuscript's clarity, methodological transparency, and the contextualization of its findings before it is ready for publication.

**Response:** Thank you for your time and effort.

**1. Abstract
1.1 The abstract states, "Within two years, most of the 2.1 million inhabitants were displaced and more than 69 500 people were directly killed." The timeline in the main text (page 2) clarifies the war started in October 2023 and the study was conducted from January to April 2025. Can you please specify the exact dates for the "two years" mentioned in the abstract to ensure consistency with the detailed timeline provided in the manuscript?**

Response: Thank you for the note. The two years we refer to are from October 2023 until the time of writing this manuscript (October 2025). We have clarified this in the abstract. (Page 2, line 40). **Modified.**

**1.2 In the abstract, you mention "three additional themes." Please ensure the three themes listed in the abstract ("Unprotected...", "Professional role...", "Challenges and potentials...") exactly match the themes as they appear in the results section of the main text for consistency.**

Response: We have revised the themes throughout the manuscript. They appear the same in the abstract, in the results section, and in Figure 1. **No modifications were needed.**

**2. Introduction
2.1 On page 2, you cite that "more than 69 500 people were directly killed, whom more than 70% are women and children." The data source (Jamaluddine et al., 2025) is a capture-recapture analysis. To enhance the scientific robustness of this statistic in the introduction, could you briefly add a note on the methodology used to arrive at this figure (e.g., "using a capture-recapture analysis, which accounts for underreporting...")?**

Response: Thank you for this query. We are referring to the figure reported in the OCHA report for November 2025 (69,513) (Reference below and cited). OCHA cites this number as reported by the Ministry of Health (MoH), which can be considered a trusted source. However, this figure is likely an underestimate, as many individuals remain unaccounted for under the rubble.

Reference: OCHA. Humanitarian Situation Update #342 | Gaza Strip: OCHA; Nov 2025 [cited 2025 7/12/2025]. Available from: <https://www.ochaopt.org/content/humanitarian-situation-update-342-gaza-strip>. **No modifications were needed.**

**2.2 On page 4, you state that "IHL... are meant to protect civilians and health care providers during war." Given the extensive documentation in your paper of attacks on healthcare, including the targeting of hospitals and the displacement of midwives, do you think a brief, explicit statement about the perceived failure of IHL in this specific context would strengthen the argument for why this study is urgently needed?**

Response: Thank you for the comment. We added this sentence:

*“This study is crucial as it generates context-specific evidence on delivering childbirth care in extreme and resource-constrained conflict settings, where formal health systems are disrupted”*. (Page 5, lines 149-151). **We hope this is satisfactory.** **Modified.**

**3. Material and Methods
3.1 In page 5: You mention "no personally identifiable information was recorded." However, you also state interviews were conducted "face-to-face." How did you ensure that the location and time of the interview, as well as the participants' visible characteristics, did not inadvertently compromise their anonymity, especially given the small sample size and the close-knit, displaced community context?**

Response: Thank you for this note. It is correct that some participants were identified by the interviewer (second author), who is a midwife residing in Gaza, and that the interviews were conducted face-to-face. However, no personal identifying information was collected or recorded during the interviews. For example, no names, phone numbers, identification numbers, or places of residence were mentioned in the recordings.

This ensured that the data remained anonymous to the other two authors. Additionally, many participants were not previously known to the interviewer prior to recruitment. We hope this clarification helps to contextualize the process and address the reviewer’s concern. **No modifications were needed.**

**3.2 In page 6: You used Perplexity AI to translate the transcripts for the third author. This is a novel approach. To ensure methodological transparency and address potential concerns about accuracy or bias, could you please elaborate on how the translated English transcripts were validated against the original Arabic? You mention they were "confirmed by the co-authors," but a more detailed step (e.g., "the two Arabic-speaking authors reviewed and approved the translated transcripts for accuracy and contextual meaning before the third author began analysis") would strengthen this section.**

Response: Thank you for the suggestion.

The sentence *“The two Arabic-speaking authors reviewed and approved the translated transcripts for accuracy and contextual meaning before the third author initiated the analysis.”* Was added. (Page 7, line 225-227). **Modified.**

**3.3 Table 1 (page 10): The table lists years of experience, but the narrative text mentions an average of \(11.0 \pm 5.93\) years. For Midwife 9, the table lists 28 years, which appears to be a significant outlier. Is this correct? If so, its impact on the analysis and findings should be noted. Also, Midwife 2 is listed as having 5 years of experience, but her narrative (MW2) is particularly rich and central to the overarching theme. It would be useful to briefly note in the text that participants spanned a wide range of experience, which contributed to the richness of the data.**

Response: Thank you for the important comment. Upon our review of the original data, the participant reported 28 years of experience; however, she also stated that she graduated in 1999 and has been working since then, which corresponds to 26 years of experience as a midwife. This has been revised and corrected in both the table and the text.

A sentence was modified as follows:

*“The midwives’ experience varied widely (mean ± SD: 12.67 ± 7.48 years), contributing to the richness of the data.”* (Page 9, lines 272-273). **Modified.**

**4. Results
4.1 Theme 1, subtheme "Improvised Childbirth" (page 11): MW9's story of using a blanket from a vegetable cart and a donkey cart for transport is incredibly powerful. Did any midwife recount a situation where improvisation failed, leading to a poor outcome (e.g., maternal or neonatal death, infection)? While your findings focus on successful outcomes, acknowledging the presence or absence of such stories is important for a complete picture. You mention this as a limitation, but a note in the results about the nature of the narratives (e.g., "All narratives shared by the midwives concluded with a live birth, though complications were managed") would be helpful.**

Response: Thank you for this important note. We (as a research team) also reflected on why midwives did not share situations involving complications/unsuccessful outcomes. We believe that midwives may have been reluctant to disclose such cases, as assisting women to deliver outside the hospital is considered an illegal practice, and midwives are not permitted to provide this type of care. Consequently, they may have feared potential repercussions or sanctions from the health system. It is therefore possible that midwives selectively shared narratives that ended without complications.

Notably, two midwives reported assisting a labouring woman with a history of infertility and a previous cesarean section, as the war was intense and both cases had no access to an ambulance to be transferred to the hospital. We did include relevant quotations from midwives (MW7 and MW8; page 18, lines 546–551, and page 19, lines 556-558), who reported assisting women with potential risks. Similarly, MW8 reported assisting a woman with a prior history of postpartum hemorrhage at a focal medical point in a school, where she was the most qualified provider and ambulance access was not possible. In this situation, Pitocin was administered intravenously, as it was available at the site.

We also acknowledged this in the limitations section (page 21, lines 659-662) and on (page 21, line 662-666), we added the following:

*“Additionally, midwives may have been reluctant to disclose cases with complications, as assisting women to deliver outside the hospital is considered an illegal practice. Consequently, they may have feared potential consequences or sanctions from the health system. It is therefore possible that midwives selectively shared narratives that ended without complications.”*

**We hope this elaboration and modification satisfy the reviewer’s comment. Modified.**

**4.2 Theme 2, subtheme "Improvising preparedness" (page 13): The quote about using a kitchen knife and sewing thread is stark. You mention that after some incidents, there was a focus on ensuring medical points had delivery kits. Can you provide more detail? What was the nature of this "focus"? Was it a formal initiative by the Ministry of Health, UNRWA, or a grassroots effort among the midwives themselves? This small detail adds significant depth to the theme of "challenges and potentials."**

Response: From midwives’ reports, it was evident that not all were provided with delivery kits. These kits are disposable and are usually supplied by international organizations such as UNFPA or local NGOs. Access to these kits appeared to depend on availability, distribution channels, and local contacts within different areas. As a result, many midwives did not receive any kits, while a few reported receiving one or two, which were subsequently used.

Focal points were established within displaced communities through grassroots community efforts. However, some of these focal points did not include midwives, as even during the most critical periods, midwives were still required to attend hospital shifts and perform their duties. All midwives reported that the cases they assisted within displaced communities occurred either outside their official working hours or after completing their hospital shifts. This was reported on page 14, lines 421-433. We believe that sufficient quotations and elaboration have been provided to support this theme. **No modifications were needed.**

**4.3 Theme 3, subtheme "Navigating high-risk pregnancies" (page 16): The quote from MW8 about the woman with a previous C-section and a "precious baby" after infertility highlights the immense pressure on midwives. Was there any instance where a midwife had to make a critical decision, such as deciding not to attempt a home birth for a clearly high-risk patient, and if so, how did they manage that situation without access to a hospital? Exploring the decision-making process in these extreme scenarios would add further depth.**

Response: Thank you for this important note. As previously indicated, midwives may have been reluctant to disclose high-risk cases due to fear of potential sanctions from the health system, as assisting women to deliver outside hospital settings is considered an illegal practice. Moreover, despite recurrent conflicts in Gaza, there is no clear strategy to guide midwives on how to provide care during such situations.

We did include relevant quotations from midwives (MW7 and MW8; page 18, lines 546–558), who reported assisting women with potential risks. In these cases, midwives were both ethically and practically compelled to provide care. For example, one woman with a history of infertility and a highly valued pregnancy; was in advanced labour (7 cm cervical dilation) during intense hostilities, with no accessible hospital for referral. In another case, MW7 assisted a woman with a previous cesarean section. Similarly, MW8 reported assisting a woman with a prior history of postpartum hemorrhage at a focal medical point in a school, where she was the most qualified provider and ambulance access was not possible. In this situation, Pitocin was administered intravenously, as it was available at the site.

It is also possible that additional high-risk cases were not reported, particularly given that the interviewer is a senior midwife and supervisor in Gaza, which may have influenced participants’ willingness to disclose such experiences due to concerns about accountability or punishment.

This was acknowledged in the limitation.

We addressed this in the strengths and limitations section. (page 21, line 662-666).

**We hope this elaboration satisfies the reviewer's query.**

**5. Discussion
5.1 Page 18: You state that "midwives during the recent war did not mention fear of restrictions, which might imply a growing recognition of the profession's autonomy." This is a crucial point. Could it also be that the complete breakdown of the formal health system and the state of emergency simply rendered legal restrictions moot? The authors might explore this nuance further: was the absence of fear due to autonomy or anarchy? This distinction has significant implications for how preparedness policies are framed (i.e., empowering vs. emergency protocols).**

Response: Thank you for this important point. Although Gaza has experienced several wars over the past two decades, this conflict has been markedly more intense and destructive. The Palestinian health system (across the West Bank and Gaza) operates within a clear hierarchical structure and is led by physicians, with established national policies stating that midwives are not permitted to assist childbirth outside hospital settings and that all women should access hospitals to give birth. This creates a clear paradox in a context of recurrent conflict. We have previously discussed these policy and practice tensions in earlier work (Wick L, Hassan S. No safe place for childbirth: women and midwives bearing witness, Gaza 2008-09. Reprod Health Matters. 2012 Dec;20(40):7-15. doi: 10.1016/S0968-8080(12)40648-6. PMID: 23245403.

While the health system was largely non-functional during this period, midwives’ accounts indicate that both midwives and women were under extreme conditions, leaving little opportunity to consider formal protocols or strategies. In many cases, women sought help from midwives known to them within displaced communities, and midwives felt compelled to respond despite the lack of resources or formal authorization. As reflected in the data, many midwives provided care without adequate equipment, not as a matter of choice, but due to the urgency and constraints of the situation.

We interpret these actions as reflecting a profound sense of professional and ethical responsibility under life-threatening conditions. Providing care while simultaneously facing personal risk of death underscores the extraordinary challenges midwives endured. We have tried to present this complexity and depth of experience through participants’ quotations and in the discussion.

**We hope this elaboration satisfies the reviewer's query.** **No modifications were needed.**

**5.2 Page 18-19: You reference the Quality Maternal and Newborn Care (QMNC) framework to highlight how midwives' care aligned with its philosophy. The most urgent missing quality was "sufficient for first line management of complications." This is a critical gap. In your discussion, could you elaborate on what specific "first-line" supplies (e.g., misoprostol for PPH, antibiotics, magnesium sulfate) were most urgently needed and how the absence of these impacted the midwives' ability to manage the high-risk cases you described? This would make the recommendation for "necessary supplies" in the conclusion more specific and actionable.**

Response: Thank you for the comment, and we agree with you. Some shelters included essential medications and supplies for the displaced population, such as intravenous cannulas, fluids, commonly used analgesics, and antibiotics. Pitocin was not available in these shelters or focal points. Some midwives were themselves living in these shelters and provided care to pregnant women as well as other patients as needed and supported by UNRWA / other local NGOs. In cases of labour, a midwife reported administering Pitocin for a woman with a history of postpartum hemorrhage, which could be available by chance with her via UNRWA/other NGOs leading such focal points in displaced communities. However, essential medications such as Pitocin and magnesium sulfate were not available in shelters. Overall, midwives working in displaced communities reported significant shortages of supplies and medications. To clarify this in the discussion section:

We have added the following sentences to the manuscript (Page 20, lines 618–622*): “Some focal medical points in shelters included essential medications and supplies for the displaced population, such as intravenous cannulas, fluids, commonly used analgesics and antibiotics. Essential medications for obstetric care, such as Pitocin and magnesium sulfate, were not available in shelters or focal medical points.”*

Additionally, we added the phrase (page 21, lines 624-625): “*except for the absence of first-line essential drugs required for the management of obstetric emergencies*.” at the end of the sentence on page 21, line 622: “Despite the alien environment, midwives’ care…”.

**We hope this clarification and revision address the reviewer’s comment.** **Modified.**

**5.3 Page 20: You mention the UNRWA's role and its being targeted. This is a critical part of the context. Could you briefly expand on how the targeting of UNRWA and the subsequent disruption of its supply chains directly affected the midwives you interviewed? Did they mention a specific point at which they lost access to UNRWA supplies? This would strengthen the link between geopolitical events and the on-the-ground experiences of your participants.**

Response: UNRWA is a main healthcare provider for Palestinian refugees, particularly in Gaza, where the majority of the population are refugees. During the war, UNRWA continued to deliver care through its health centers (except when there is a call for evacuation) and selected shelters. A few midwives reported working in focal medical points established within shelters (often schools), which were equipped with basic supplies by UNRWA; including delivery kits. Delivery kits were mainly assembled and distributed by UNFPA and other national non-governmental organizations to support midwives and women in displaced communities.

During the war, all health sectors experienced severe shortages of supplies due to the high number of injuries and the complexity of care required. The midwives interviewed primarily described their work with pregnant women in displaced communities. Many of these midwives were formally employed in hospitals but provided care outside their official working hours, as they were accessible to women in their communities. A smaller number worked within focal medical points in shelters supported by UNRWA and/or other NGOs.

For clarification, we expanded the sentence on page 21, lines 643–644 to read as follows:

*“The UN Relief and Works Agency for Palestine Refugees in the Near East (UNRWA) provided a well-organized system for involving midwives in preparedness and response within the health system during crises (2), which continued to provide services for communities and support some midwives in focal medical points in shelters.”*

**We hope this clarification and revision satisfy the reviewer’s comments.** **Modified.**

**6. Strengths and Limitations
6.1 Page 20: The stated limitation is that "all participating midwives shared stories that, in some way, ended well with a healthy mother and baby. Important information may have refrained from participating due to fear of being perceived as having failed." This is an important and honest limitation. Could the authors expand on this by discussing the potential impact of this selection bias on the findings? For instance, does this mean the findings represent a "best-case scenario" of midwifery response, and that the experiences of those who faced tragic outcomes might reveal different, perhaps even more critical, failures in the system? Acknowledging this gap explicitly would be valuable.**

Response: We have elaborated on this point in response to previous comments. We have also addressed this in the limitations (page 21, line 662-666), we added the following:

*“Additionally, midwives may have been reluctant to disclose cases with complications, as assisting women to deliver outside the hospital is considered an illegal practice. Consequently, they may have feared potential consequences or sanctions from the health system. It is therefore possible that midwives selectively shared narratives that ended without complications.”*

**We hope this elaboration and modification satisfy the reviewer’s comment. Modified.**

**7. Conclusion and Recommendations
7.1 Based on your findings, what would be the first, most critical element of such a policy? Is it the provision of a standardized "emergency birth kit" with essential supplies? Is it a legal framework that explicitly authorizes and protects midwives practicing in community settings during declared emergencies? Prioritizing this in the final sentence of your conclusion could give your recommendation more immediate impact.**

Response: Thank you for this inquiry. We revised the conclusion section, and priorities are as presented. The primary priority is the development of clear policies aligned with midwives’ scope of practice that support their professional role when caring for pregnant women in war. This is particularly critical in contexts such as Palestine, where the situation has remained politically unstable and has experienced prolonged conflict for over 70 years due to occupation (page 22, lines 680–684).

The second priority is strengthening the preparedness of midwives to effectively assist women outside hospital settings during crises (page 22, lines 685–686). Finally, ensuring the availability of adequate supplies and resources is essential to enable midwives to provide safe and effective care during emergencies (page 22, lines 687–688).

The Conclusion section already reflects these priorities, and therefore, **no modifications were needed.**

**END OF RESPONSE TO REVIEWERS’ COMMENTS**
